# PBX3 in Cancer

**DOI:** 10.3390/cancers12020431

**Published:** 2020-02-13

**Authors:** Richard Morgan, Hardev S Pandha

**Affiliations:** 1 Institute of Cancer Therapeutics, Faculty of Life Sciences, University of Bradford, Bradford BD7 1DP, UK; 2 Faculty of Health and Medical Sciences, University of Surrey, Guildford GU2 7XH, UK; h.pandha@surrey.ac.uk

**Keywords:** PBX3, PBX, HOX, microRNA, acute myeloid leukemia, gastric cancer, colorectal cancer, liver cancer

## Abstract

PBX3 is a homeodomain-containing transcription factor of the pre-B cell leukemia (PBX) family, members of which have extensive roles in early development and some adult processes. A number of features distinguish PBX3 from other PBX proteins, including the ability to form specific and stable interactions with DNA in the absence of cofactors. PBX3 has frequently been reported as having a role in the development and maintenance of a malignant phenotype, and high levels of PBX3 tumor expression have been linked to shorter overall survival in cancer. In this review we consider the similarities and differences in the function of PBX3 in different cancer types and draw together the core signaling pathways involved to help provide a better insight into its potential as a therapeutic target.

## 1. Introduction

The pre-B cell leukemia (PBX) family is a group of homeodomain-containing transcription factors and homologues of the *Drosophila Extradenticle* gene [1]. Humans have 4 PBX homologues, PBX1–4, all of which encode a protein that contains a homeodomain DNA-binding region and a protein interaction domain (PBC) that facilities interaction with PBX cofactors, including Myeloid Ecotropic Viral Integration Site 1 Homolog (MEIS) and HOX proteins [1]. PBX3 differs from other PBX proteins, as it can form a stable interaction with DNA as a monomer or homodimer, with a consensus binding sequence of TGATTGATTTGAT [2]. The other PBX paralogues bind to only a subset of this, TGATTTAT, and the interaction requires binding of a HOX protein of the *Annetenapedia* family (i.e., HOX paralogues 1-9) [2].

PBX proteins were initially and most extensively characterized for their role in early development, especially anteroposterior patterning of the main body axis and the limbs through forming heterodimers with HOX proteins, which are themselves expressed in a spatial order along this axis [3]. Many of these studies identify either PBX1 or PBX2 as HOX-binding partners, and PBX3 [4], which was characterized relatively late, has had very few specific developmental roles ascribed to it. The most notable of these is a role in maintaining the undifferentiated state of embryonic stem cells [5], although it also has a potential role in cardiac development and congenital cardiac defects in humans [6]. To date, PBX3 is more frequently associated with cancer, and has been reported to be overexpressed in many solid tumors, as well as in several hematological malignancies, where it has a role in promoting cell survival, invasion, and proliferation. Here, we review the molecular mechanisms underlying these oncogenic functions in different cancers, and consider the potential of PBX3 as a therapeutic target.

## 2. Transcriptional and Post-transcriptional Regulation of PBX3

PBX genes, including PBX3, were originally shown to be regulated by retinoic acid in P19 embryonic stem cells [7], and a subsequent study revealed that this is dependent on retinoic acid receptor alpha (RARα) and occurs both at the level of transcription (although indirectly), and at the protein level, as the stability of PBX proteins is significantly increased (from approximately 6 hours to 12 hours) after treatment [8]. While this mode of regulation seems to be more relevant to early developmental processes [9], a number of other mechanisms have emerged as potential, specific regulators of PBX3 in the context of cancer. These include histone methylation in promoter and enhancer regions of PBX3 [10,11], as well as DNA methylation as revealed by a significant increase in PBX hypomethylation in CBFP-MYH11-rearranged acute myeloid leukemia (AML) [12]. There is also evidence for the regulation of PBX3 expression by androgen signaling in prostate cancer [13,14]. However, by far, the most frequently described mechanism of PBX3 regulation is the post-transcriptional inhibition through microRNAs (miRs). These are small (around 22 bases) non-coding RNA transcripts that can post-transcriptionally regulate gene expression by forming duplexes with mRNAs to which they are complementary. The formation of such a duplex can result in RNA cleavage, destabilization of the mRNA through shortening of the polyA tail, and/or direct inhibition of translation. It is now recognized that miRs play a significant role in regulating gene expression, with around 1900 miRs having been identified in the human genome [15]. Furthermore, miR-mediated regulation of gene expression seems to be of particular relevance in cancer, as many of the miRs identified to date have tumor suppressor functions [16].

The first report of a PBX3-specific miR was published in 2011, when Ramberg et al. demonstrated that the miR-let-7d repressed PBX3 expression in prostate cancer [14], and this was followed shortly after by a report that the closely related miR-let-7c was complementary to the 3’UTR of PBX3 and could directly repress its expression in colorectal cancer [17], as could miR-let-7b in glioma [18]. Subsequently, PBX3 expression was also shown to be reduced by miR-181 in AML [19], miR-129-5p in pancreatic cancer [20], miR-495 in AML [21] and melanoma [22], miR-200b in breast cancer [23], miR-200b, miR-222, and miR-424 in hepatocellular carcinoma (HCC) [24], miR-320a in multiple myeloma [25], gastric cancer [26], and cancer-associated fibroblasts (CAFs) associated with HCC [27], miR-33a-3p in HCC [28], miR-497 in multiple myeloma [29], miR-144-3p in gastric cancer [30], miR-98 in glioma [31], miR-144 in lung cancer [32] and HCC [33], miR-302a in HCC [34], and miR-526b in cervical cancer [35]. In addition, within the context of early development, miR-320 was shown to maintain the undifferentiated state in chick blastodermal cells through repression of PBX3 [5]. All of these miRs were shown to bind to the 3’UTR of PBX3, along with a number of other target transcripts in some cases (Table 1).

### Acute Myeloid Leukemia

The role of PBX3 has been most extensively investigated in AML. Along with a number of homeobox-containing transcription factors, PBX3 has been shown to be an oncogene in this malignancy. Forced overexpression of PBX3 along with its cofactor MEIS can transform normal hematopoietic stem cells in mice, leading to the formation of AML with a latency period similar to that observed for MLL-AF9, the most commonly observed oncogenic gene fusion, and a corresponding upregulation of HOXA cluster genes [36]. This concurs with the findings of more recent studies identifying PBX3 as a key transcriptional regulator of HOXA genes [37], and promoter of cell proliferation and resistance to chemotherapeutic agents [38]. PBX3 knockdown in both OCI-AML3 and U937 cells was shown to dramatically increase their sensitivity to cytarabine, and to a lesser extent mylotarg (a drug-antibody conjugate targeting CD33 [39]) [38]. A similar approach using a mouse model of leukemia development revealed that this oncogenic role was specific to PBX3, as neither PBX1 nor PBX2 could substitute for it in forced expression experiments [40]. PBX3 binding to MEIS was shown to significantly increase the stability of the latter as, in the absence of PBX3, MEIS was rapidly degraded in a proteasome-dependent manner [41].

MEIS and PBX3 are necessary for the formation of stable high-affinity DNA/HOXA9/PBX3/MEIS complexes that in turn activate the transcription of key downstream targets, including FMS-like tyrosine kinase 3 (Flt3) and Tribbles 2 (Trib2) [41]. Both Flt3 and Trib2 have been characterized as oncogenes in AML, as well as in many solid malignancies. Flt3 is a tyrosine kinase receptor kinase that promotes proliferation and survival of hematopoietic stem cells and is one of the most frequently mutated genes in cytogenetically normal AML, with activating mutations being sufficient to transform 32D cells [42]. Trib2 is a pseudokinase with a very wide interactome, including components of pro-oncogenic signaling pathways, such as AKT [43]. High levels of Trib2 expression in AML are associated with drug resistance due to upregulation of AKT signaling and a consequent increase in cell proliferation, as well as increased cell survival mediated in part by elevated Bcl-2 expression [44].

Other studies have also supported a pro-oncogenic role of PBX3 in AML. For example, in NUP98-HOXD13-transformed AML, PBX3 was found to be necessary for the continued proliferation and survival of malignant cells [45], and suppression of PBX3 transcription through inhibition of the H3K79 methylase blocked the proliferation of NPM1-driven leukemia [11].

As discussed above, two miRs that target PBX3 at the post-transcriptional level both have tumor suppressor functions in AML [19,21]. miR-181 was shown to block the proliferation and survival of AML cells both in vitro and in vivo, with a significantly longer latency in a mouse model of this disease. Furthermore, effects of the elevated miR-181 expression could be partially rescued through PBX3 overexpression [19]. Likewise, miR-495 can also directly target PBX3 through binding to the 3’UTR of the transcript, and in doing so inhibits cell proliferation—an effect that can also be blocked by higher expression of PBX3 [21].

These in vivo and in vitro findings are further supported by clinical observations from multiple datasets that elevated PBX3 expression in patients with NMP1-mutated AML is associated with shorter overall survival [46].

## 3. Gastric Cancer

Elevated PBX3 tumor expression has been found to be associated with a number of key clinical and pathological indicators associated with a poor outcome in gastric cancer, including invasion depth, and the stage and grad e of the tumor [47,48], and, correspondingly, tumor expression of miR-144-3p, which blocks PBX3 expression at the post-transcriptional level, is negatively correlated with tumor stage, invasion depth, and nodal metastasis [30]. More recently, miR-320a was also shown to have a tumor-suppressive role in gastric cancer through targeting PBX3, and that the expression of this miR in gastric cancer was repressed in part by methylation of its promoter [26].

In vitro studies have indicated a role for PBX3 in promoting epithelial-to-mesenchymal transition (EMT) [30,49], which in turn enables invasion and metastasis through a reduction in cellular adherence and an increase in migration. This may be partly dependent on activation of the AKT pathway, as there was a significant increase in phosphorylated AKT (Ser473) in the cells overexpressing PBX3 [49]. The overexpression of PBX3 also resulted in an increase in MMP9 activity [49], a key protease involved in metastasis [50], as well as an increase in the ability of gastric cancer cells to promote tubule formation by HUVEC cells, indicating an increase in pro-angiogenic signaling [49].

## 4. Colorectal Cancer

The first indication of a role of PBX3 in colorectal cancer was the finding that lower tumor expression of the miR-let-7c was associated with increased metastases, increased grade, and shorter survival. The same study demonstrated that miR-let-7c targets PBX3, as well as K-Ras and MMP11, in the colorectal cancer-derived cell line LoVo [17]. Overexpression of PBX3 without its 3’UTR-let-7c-binding sequence was able to rescue cells from tumor suppressive effects of this miR [17]. These findings are in agreement with those of the study looking at PBX3 RNA expression in colorectal tumors, in which high expression levels were found to be significantly associated with lymph node invasion, metastasis, advanced pathological stage, and shorter overall survival [51]. A more recent study revealed that PBX3 is highly expressed in the cells characterized by high levels of the WNT signaling activity at the edge of colorectal tumors, and that PBX3 expression in cells is dependent on the WNT signaling as demonstrated by knockdown of the key mediator of the WNT pathway, beta-catenin. PBX3 was also shown to be necessary for EMT in colorectal cancer cells, and is upregulated by the SNAIL and Zeb1 EMT-associated transcription factors, possibly through an indirect mechanism involving suppression of the PBX3-targeting miR-200c [52].

## 5. Liver Cancer

Tumor-initiating cells (TICs) are a subset of cancer cells that have exceptionally high tumorigenicity and often also display resistance to chemotherapy and radiotherapy, and are thus of particular importance as targets in cancer [53]. In hepatic cellular carcinoma (HCC), a subpopulation of cells that express voltage-gated calcium channel α2δ1 have been shown to have TIC-like properties, and also to express PBX3 at a high level [24]. Repression of PBX3 activity by 4 miRs (miR-let-7c, miR-200b, miR-222, miR-424) in non-TICs is sufficient to block the TIC phenotype, and PBX3 was shown to activate the transcription of α2δ1, as well as other genes involved in the maintenance of the stem-cell phonotype, such as SOX2 and SALL2 [24]. A subsequent study showed that another miR, miR-33a-3p, also blocks PBX3 expression in HCC cells and results in a reduction in invasion and metastasis, which concurs with clinical data for primary HCC demonstrating that low levels of miR-33a-3p expression are associated with a greater risk of metastasis and shorter overall survival [28]. There is also evidence that an additional miR, miR-320a, can be transferred to HCC cells from neighboring cancer-associated fibroblasts (CAFs) via exosomes. Once in HCC cells, miR-320a can block proliferation and migration through inhibition of EMT, as well as cyclin-dependent kinase 2 (CDK2) and MMP2 [27], and (from a later study) MAP3K2 [34].

## 6. Glioma

Similar findings to those described for other cancers have also been obtained for glioma. Silencing PBX3 in glioma cells has been shown to reduce proliferation both in vitro and in vivo [54], and targeting of PBX3 by miR-98 reduced invasion and migration of glioma cells in an orthotopic model [18]. Cell proliferation could be blocked and apoptosis induced through miR-320, which targets PBX3 and consequently Raf1/MAPK1 pathway activation [55]. PBX3 also promotes a mesenchymal phenotype in glioma cells through a positive feedback pathway involving MEK, ERK1/2, LIN28, and miR-let-7b. Ectopic expression of PBX3 activated MEK/ERK1/2 signaling, upregulating a potent oncogenic transcription factor that promotes cell proliferation and survival, and can also immortalize cells through increasing telomerase activity [56]. cMyc can also activate LIN28 expression, which in turn inhibits miR-let-7b transcription, de-repressing genes that promote invasion such as IL-6 and HMGA2 [18]. To complete this cycle, miR-let-7b can post-transcriptionally repress PBX3, thus PBX3 expression can begin a positive feedback, increasing its own expression in glioma cells [18].

## 7. Other Cancers

PBX3 expression has been reported in a number of other solid malignancies and in general is associated with a poor prognosis. This includes prostate cancer, in which PBX3 is expressed at higher levels in malignant compared to benign diseases, with the androgen-regulated miR inhibitor of PBX3, miR-let-7d, showing the opposite trend [14]. Notably, PBX3 seems to increase in expression within prostate glands that are adapting to androgen deprivation, which would correspond to reduced expression of miR-let-7d [13].

In cervical cancer, PBX3 overexpression promotes proliferation through the AKT pathway, and high levels of expression in primary tumors are associated with a poor prognosis [57], and, as with the other cancers described above, PBX3 expression is suppressed by an miR, miR-526b, preventing cells from undergoing EMT [35].

Other malignances in which miRs have been shown to promote apoptosis and block proliferation through targeting PBX3 are multiple myeloma [25,29], melanoma [22], breast cancer [23], pancreatic cancer [20], and lung cancer [32].

## 8. The EWSR1-PBX3 Translocation

EWS RNA-binding protein 1 (EWSR1) encodes a multifunctional protein involved in a range of cellular processes, including cell signaling, RNA processing and transport, and gene expression. It is frequently involved in oncogenic translocations, best characterized in Ewing’s sarcoma, and it is usually the transcriptional activation domain of EWSR1 that forms a chimera with the DNA-binding domain of the partner gene. EWSR1–PBX3 gene fusions have been identified in myoepithelial tumors of bone and soft tissue [58,59,60], and subsequently in retroperitoneal leiomyoma [59]. The majority of these fusions involve a break at exon 8 in EWSR1 and exon 5 of PBX3, but in all cases result in the transcriptional activator domain of EWSR1 fusing to the homeodomain of PBX3. Intriguingly, although EWSR1–PBX3 is a relatively rare translocation even within the chimeric EWSR1 family, it is very common in cutaneous syncytial myoepithelioma, although the reasons for this selectivity remain unclear [58].

## 9. PBX3-regulated Pathways in Cancer

**Figure 1:**
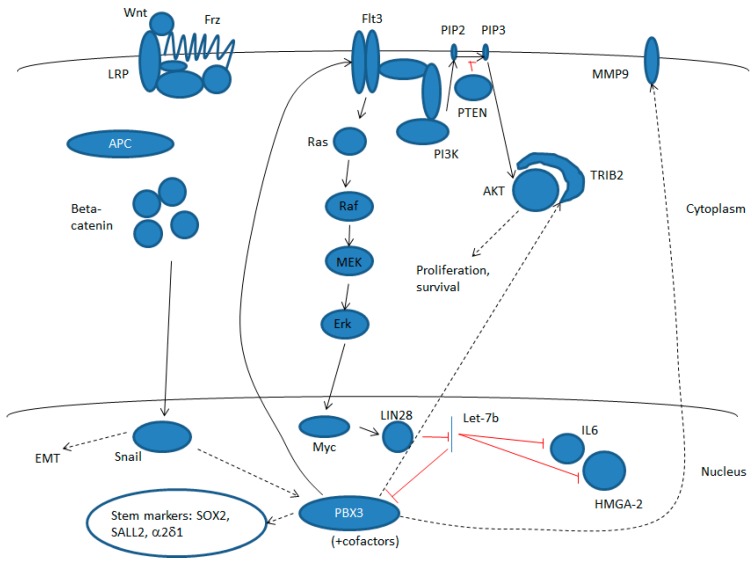
Pre-B cell leukemia (PBX3) is a target and a regulator of multiple signaling pathways. These include the MEK/ERK pathway, and notably tyrosine kinase receptor Flt3. Activation of this pathway represents a positive feedback loop in which increased expression of the Myc transcription factor activates LIN28 expression, which in turn inhibits biogenesis of miR-let-7b, an miR that blocks PBX3 expression post-transcriptionally. PBX3 also increases signaling through the AKT pathway by activating expression of the AKT activator protein, TRIP2. In addition, PBX3 expression increases in response to the signaling through the canonical WNT pathway via the activation of the Snail transcription factor. Activation of PBX3 through these pathways increases epithelial-to-mesenchymal transition (EMT) and hence invasion and metastasis (in part through increasing MMP9, IL6, and HMGA-2 expression) as well as cell survival and proliferation, and can also confer a TIC phenotype through upregulation of the SOX2 and SALL2 transcription factors and voltage-gated calcium channel α2δ1. Dashed lines represent indirect pathways involving multiple steps and additional components.

Based on the above, it is becoming clear that PBX3 interacts principally with the MAPK, AKT, and WNT signaling pathways (Figure 1). PBX3 was shown to increase signaling through MEK/ERK in several studies [18,57,58], although to date the only component of the pathway shown to be directly upregulated by PBX3 is the tyrosine kinase receptor Flt3 [41]. Activation of this pathway represents a positive feedback loop in which increased expression of the Myc transcription factor activates LIN28 expression, which in turn inhibits biogenesis of miR-let-7b, an miR that blocks PBX3 expression post-transcriptionally [18]. PBX3 also increases signaling through the AKT pathway, and an increase in phosphorylated AKT has been demonstrated upon ectopic PBX3 expression [49], which may be the result, at least in part, of a PBX3-mediated increase in TRIP2 expression [41]. TRIP2 binds to AKT and promotes its phosphorylation at Ser473 [61]. PBX3 expression also increases in response to the signaling through the canonical WNT pathway via the activation of the Snail transcription factor [52]. Activation of PBX3 through these pathways increases EMT and hence invasion and metastasis (in part through increasing MMP9 [49], IL6, and HMGA-2 [18] expression) as well as cell survival and proliferation, and can also confer a TIC phenotype through upregulation of the SOX2 and SALL2 transcription factors and the voltage-gated calcium channel α2δ1 [24]. Hence, PBX3 is both a target and a regulator of the three key signaling pathways involved in formation and maintenance of the malignant phenotype, and consequently also interacts, at least indirectly, with multiple oncogenes.

## 10. Targeting PBX3

The clear role emerging for PBX3 as a promoter of cell survival, invasion, and metastasis in cancer makes it an attractive therapeutic target. However, conventionally it has been difficult to directly target transcription factors due to their intracellular location and their tendency to interact through large, hydrophobic surfaces. One approach to targeting PBX in general has been the use of cell-penetrating peptides that disrupt binding between PBX proteins and their HOX cofactors. These peptides have been shown to trigger apoptosis in a wide range of cancers [62]. However, this approach is not specific to PBX3.

Other approaches have included indirect targeting at the level of transcription through altering methylation of enhancer and promoter regions. As described above, inhibition of DOT1L, a H3K79 methyltransferase, reduced PBX3 expression in AML cells and induced apoptosis, although this might be an indirect mechanism through HOXA9 [11].

It may also be possible to exploit the extensive network of miRs that target PBX3. Synthetic miRs (also referred to as miR mimics) are currently in clinical trials for cardiovascular diseases [63], although for cancer only two miR mimics have entered trials to date, and of these only TargomiR, which is a mimic of miR-16, is likely to progress beyond Phase I [64]. It may also be possible to develop small molecule inhibitors of PBX3 that do not target cofactor or DNA binding, but instead target PBX3 for degradation in the proteasome using the E3-ligase targeting (PROTAC) technology [65].

## 11. Conclusions

PBX3 is emerging as a functionally significant transcription factor in a range of cancers, and in the majority of these its expression is linked to aggressive disease and shorter overall survival. It is both a regulator of and regulated by the MEK/ERK, WNT, and AKT signaling pathways and supports an oncogenic phenotype by promoting EMT, stem-like properties, and survival and proliferation. Its regulation by multiple miRs that are in turn key tumor suppressors indicates that it could be an important target in cancer.

## Figures and Tables

**Table 1:** PBX3-targetting microRNAs (miRs) in cancer. AML, acute myeloid leukemia; HCC, hepatocellular carcinoma; lncRNA, long non-coding RNA; MAP3K2, mitogen-activated protein kinase kinase2; MM, multiple myeloma; MMP11, matrix metalloprotease 11; UTR, untranslated region.

miR	Cancers	Binding/Other Targets/Regulation	Ref.
miR-let-7d	Prostate cancer	Upregulated by androgen	[14]
miR-let-7c	Colorectal cancer	Binds 3’UTR, also targets MMP11	[17]
miR-let-7b	Glioma	Binds 3’UTR	[18]
miR-181	AML	Binds 3’UTR	[19]
miR-129-5p	Pancreatic cancer	Binds 3’UTR	[20]
miR-495	AML and melanoma	Binds 3’UTR, also targets MEIS1	[21]
miR-200b	HCC and breast cancer	Binds 3’UTR	[23,24]
miR-222	HCC	Binds 3’UTR	[24]
miR-424	HCC	Binds 3’UTR	[24]
miR-320a	MM, gastric cancer, and HCC	Binds 3’UTR	[25,26,27]
miR-33a-3p	HCC	Binds 3’UTR	[28]
miR-497	MM	Binds 3’UTR	[29]
miR-144-3p	Gastric cancer	Binds 3’UTR	[30]
miR-98	Glioma	Binds 3’UTR	[31]
miR-144	Lung cancer, HCC	Binds 3’UTR, also binds the lncRNA UCA1	[32,33]
miR-302a	HCC	Binds 3’UTR, also targets MAP3K2	[34]
miR-526b	Cervical cancer	Binds 3’UTR	[35]

## References

[1] Longobardi E., Penkov D., Mateos D., De Florian G., Torres M., Blasi F. (2014). Biochemistry of the tale transcription factors PREP, MEIS, and PBX in vertebrates. Dev. Dyn..

[2] Neuteboom S.T., Murre C. (1997). Pbx raises the DNA binding specificity but not the selectivity of antennapedia Hox proteins. Mol. Cell Biol..

[3] Mallo M., Wellik D.M., Deschamps J. (2010). Hox genes and regional patterning of the vertebrate body plan. Dev. Biol..

[4] Monica K., Galili N., Nourse J., Saltman D., Cleary M.L. (1991). PBX2 and PBX3, new homeobox genes with extensive homology to the human proto-oncogene PBX1. Mol. Cell Biol..

[5] Lee S.I., Jeon M.H., Kim J.S., Park J.K., Park E.W., Jeon I.S., Byun S.J. (2014). The miR-302 cluster transcriptionally regulated by POUV, SOX and STAT5B controls the undifferentiated state through the post-transcriptional repression of PBX3 and E2F7 in early chick development. Mol. Reprod. Dev..

[6] Farr G.H., Imani K., Pouv D., Maves L. (2018). Functional testing of a human PBX3 variant in zebrafish reveals a potential modifier role in congenital heart defects. Dis. Model Mech..

[7] Knoepfler P.S., Kamps M.P. (1997). The Pbx family of proteins is strongly upregulated by a post-transcriptional mechanism during retinoic acid-induced differentiation of P19 embryonal carcinoma cells. Mech. Dev..

[8] Qin P., Haberbusch J.M., Soprano K.J., Soprano D.R. (2004). Retinoic acid regulates the expression of PBX1, PBX2, and PBX3 in P19 cells both transcriptionally and post-translationally. J. Cell Biochem..

[9] Qin P., Cimildoro R., Kochhar D.M., Soprano K.J., Soprano D.R. (2002). PBX, MEIS, and IGF-I are potential mediators of retinoic acid-induced proximodistal limb reduction defects. Teratology.

[10] Sun H., Huang H., Li D., Zhang L., Zhang Y., Xu J., Liu Y., Liu Y., Zhao Y. (2019). PBX3 hypermethylation in peripheral blood leukocytes predicts better prognosis in colorectal cancer: A propensity score analysis. Cancer Med..

[11] Zhang W., Zhao C., Zhao J., Zhu Y., Weng X., Chen Q., Sun H., Mi J.Q., Li J., Zhu J. (2018). Inactivation of PBX3 and HOXA9 by down-regulating H3K79 methylation represses NPM1-mutated leukemic cell survival. Theranostics.

[12] Hajkova H., Fritz M.H., Haskovec C., Schwarz J., Salek C., Markova J., Krejcik Z., Dostalova Merkerova M., Kostecka A., Vostry M. (2014). CBFB-MYH11 hypomethylation signature and PBX3 differential methylation revealed by targeted bisulfite sequencing in patients with acute myeloid leukemia. J. Hematol. Oncol..

[13] Nishan U., Rosa-Ribeiro R., Cesar C.L., Carvalho H.F. (2019). Transcription regulators are transiently expressed during the prostate gland adaptation to the hypoandrogenic environment. Histol. Histopathol..

[14] Ramberg H., Alshbib A., Berge V., Svindland A., Tasken K.A. (2011). Regulation of PBX3 expression by androgen and Let-7d in prostate cancer. Mol. Cancer.

[15] Liu H., Lei C., He Q., Pan Z., Xiao D., Tao Y. (2018). Nuclear functions of mammalian MicroRNAs in gene regulation, immunity and cancer. Mol. Cancer.

[16] Shirjang S., Mansoori B., Asghari S., Duijf P.H.G., Mohammadi A., Gjerstorff M., Baradaran B. (2019). MicroRNAs in cancer cell death pathways: Apoptosis and necroptosis. Free Radic. Biol. Med..

[17] Han H.B., Gu J., Zuo H.J., Chen Z.G., Zhao W., Li M., Ji D.B., Lu Y.Y., Zhang Z.Q. (2012). Let-7c functions as a metastasis suppressor by targeting MMP11 and PBX3 in colorectal cancer. J. Pathol..

[18] Xu X., Bao Z., Liu Y., Jiang K., Zhi T., Wang D., Fan L., Liu N., Ji J. (2018). PBX3/MEK/ERK1/2/LIN28/let-7b positive feedback loop enhances mesenchymal phenotype to promote glioblastoma migration and invasion. J. Exp. Clin. Cancer Res..

[19] Li Z., Huang H., Li Y., Jiang X., Chen P., Arnovitz S., Radmacher M.D., Maharry K., Elkahloun A., Yang X. (2012). Up-regulation of a HOXA-PBX3 homeobox-gene signature following down-regulation of miR-181 is associated with adverse prognosis in patients with cytogenetically abnormal AML. Blood.

[20] Qiu Z., Wang X., Shi Y., Da M. (2019). miR-129-5p suppresses proliferation, migration, and induces apoptosis in pancreatic cancer cells by targeting PBX3. Acta Biochim. Biophys. Sin. (Shanghai).

[21] Jiang X., Huang H., Li Z., He C., Li Y., Chen P., Gurbuxani S., Arnovitz S., Hong G.M., Price C. (2012). MiR-495 is a tumor-suppressor microRNA down-regulated in MLL-rearranged leukemia. Proc. Natl. Acad. Sci. USA.

[22] Chen G., Xie Y. (2018). miR-495 inhibits proliferation, migration, and invasion and induces apoptosis via inhibiting PBX3 in melanoma cells. Onco Targets Ther..

[23] Zhao J., Meng R., Yao Q., Wang H., Niu J., Cui Y.U., Chen S., Bai Y. (2019). Long non-coding RNA HEIH promotes breast cancer development via negative modulation of microRNA-200b. Pharmazie.

[24] Han H., Du Y., Zhao W., Li S., Chen D., Zhang J., Liu J., Suo Z., Bian X., Xing B. (2015). PBX3 is targeted by multiple miRNAs and is essential for liver tumour-initiating cells. Nat. Commun..

[25] Lu Y., Wu D., Wang J., Li Y., Chai X., Kang Q. (2016). miR-320a regulates cell proliferation and apoptosis in multiple myeloma by targeting pre-B-cell leukemia transcription factor 3. Biochem. Biophys. Res. Commun..

[26] Li Y.S., Zou Y., Dai D.Q. (2019). MicroRNA-320a suppresses tumor progression by targeting PBX3 in gastric cancer and is downregulated by DNA methylation. World J. Gastrointest. Oncol..

[27] Zhang Z., Li X., Sun W., Yue S., Yang J., Li J., Ma B., Wang J., Yang X., Pu M. (2017). Loss of exosomal miR-320a from cancer-associated fibroblasts contributes to HCC proliferation and metastasis. Cancer Lett..

[28] Han S.Y., Han H.B., Tian X.Y., Sun H., Xue D., Zhao C., Jiang S.T., He X.R., Zheng W.X., Wang J. (2016). MicroRNA-33a-3p suppresses cell migration and invasion by directly targeting PBX3 in human hepatocellular carcinoma. Oncotarget.

[29] Yu T., Zhang X., Zhang L., Wang Y., Pan H., Xu Z., Pang X. (2016). MicroRNA-497 suppresses cell proliferation and induces apoptosis through targeting PBX3 in human multiple myeloma. Am. J. Cancer Res..

[30] Li B., Zhang S., Shen H., Li C. (2017). MicroRNA-144-3p suppresses gastric cancer progression by inhibiting epithelial-to-mesenchymal transition through targeting PBX3. Biochem Biophys. Res. Commun..

[31] Xu X., Bao Z., Liu Y., Ji J., Liu N. (2017). MicroRNA-98 Attenuates Cell Migration and Invasion in Glioma by Directly Targeting Pre-B Cell Leukemia Homeobox 3. Cell Mol. Neurobiol..

[32] Li D., Li H., Yang Y., Kang L. (2018). Long Noncoding RNA Urothelial Carcinoma-Associated 1 Promotes the Proliferation and Metastasis of Human Lung Tumor Cells by Regulating MicroRNA-144. Oncol. Res..

[33] Ma Y., Zhang H., Li G., Hu J., Liu X., Lin L. (2019). LncRNA ANRIL promotes cell growth, migration and invasion of hepatocellular carcinoma cells via sponging miR-144. Anticancer Drugs.

[34] Wang M., Lv G., Jiang C., Xie S., Wang G. (2019). miR-302a inhibits human HepG2 and SMMC-7721 cells proliferation and promotes apoptosis by targeting MAP3K2 and PBX3. Sci. Rep..

[35] Li H., Wang J., Xu F., Wang L., Sun G., Wang J., Yang Y. (2019). By downregulating PBX3, miR-526b suppresses the epithelial-mesenchymal transition process in cervical cancer cells. Future Oncol..

[36] Li Z., Chen P., Su R., Hu C., Li Y., Elkahloun A.G., Zuo Z., Gurbuxani S., Arnovitz S., Weng H. (2016). PBX3 and MEIS1 Cooperate in Hematopoietic Cells to Drive Acute Myeloid Leukemias Characterized by a Core Transcriptome of the MLL-Rearranged Disease. Cancer Res..

[37] Ye J., Luo D., Yu J., Zhu S. (2019). Transcriptome analysis identifies key regulators and networks in Acute myeloid leukemia. Hematology.

[38] Dickson G.J., Liberante F.G., Kettyle L.M., O’Hagan K.A., Finnegan D.P., Bullinger L., Geerts D., McMulin M.F., Lappin T.R., Mills K.L. (2013). HOXA/PBX3 knockdown impairs growth and sensitizes cytogenetically normal acute myeloid leukemia cells to chemotherapy. Haematologica.

[39] Lai C., Doucette K., Norsworthy K. (2019). Recent drug approvals for acute myeloid leukemia. J. Hematol. Oncol..

[40] Li Z., Zhang Z., Li Y., Arnovitz S., Chen P., Huang H., Jiang X., Hong G.M., Kunjamma R.B., Ren H. (2013). PBX3 is an important cofactor of HOXA9 in leukemogenesis. Blood.

[41] Garcia-Cuellar M.P., Steger J., Fuller E., Hetzner K., Slany R.K. (2015). Pbx3 and Meis1 cooperate through multiple mechanisms to support Hox-induced murine leukemia. Haematologica.

[42] Mizuki M., Fenski R., Halfter H., Matsumura I., Schmidt R., Muller C., Gruning W., Kratz-Albers K., Serve S., Steur C. (2000). Flt3 mutations from patients with acute myeloid leukemia induce transformation of 32D cells mediated by the Ras and STAT5 pathways. Blood.

[43] Zanella F., Renner O., Garcia B., Callejas S., Dopazo A., Peregrina S., Carnero A., Link W. (2010). Human TRIB2 is a repressor of FOXO that contributes to the malignant phenotype of melanoma cells. Oncogene.

[44] O’Connor C., Yalla K., Salome M., Moka H.A., Castaneda E.G., Eyers P.A., Keeshan K. (2018). Trib2 expression in granulocyte-monocyte progenitors drives a highly drug resistant acute myeloid leukaemia linked to elevated Bcl2. Oncotarget.

[45] Novak R.L., Harper D.P., Caudell D., Slape C., Beachy S.H., Aplan P.D. (2012). Gene expression profiling and candidate gene resequencing identifies pathways and mutations important for malignant transformation caused by leukemogenic fusion genes. Exp. Hematol..

[46] Nagy A., Osz A., Budczies J., Krizsan S., Szombath G., Demeter J., Bodor C., Gyorffy B. (2019). Elevated HOX gene expression in acute myeloid leukemia is associated with NPM1 mutations and poor survival. J. Adv. Res..

[47] Li Y., Sun Z., Zhu Z., Zhang J., Sun X., Xu H. (2014). PBX3 is overexpressed in gastric cancer and regulates cell proliferation. Tumour. Biol..

[48] Ma Y.Y., Zhang Y., Mou X.Z., Liu Z.C., Ru G.Q., Li E. (2017). High level of homeobox A9 and PBX homeobox 3 expression in gastric cancer correlates with poor prognosis. Oncol. Lett..

[49] Wang S., Li C., Wang W., Xing C. (2016). PBX3 promotes gastric cancer invasion and metastasis by inducing epithelial-mesenchymal transition. Oncol. Lett..

[50] Jacob A., Prekeris R. (2015). The regulation of MMP targeting to invadopodia during cancer metastasis. Front. Cell Dev. Biol..

[51] Han H.B., Gu J., Ji D.B., Li Z.W., Zhang Y., Zhao W., Wang L.M., Zhang Z.Q. (2014). PBX3 promotes migration and invasion of colorectal cancer cells via activation of MAPK/ERK signaling pathway. World J. Gastroenterol. (WJG).

[52] Lamprecht S., Kaller M., Schmidt E.M., Blaj C., Schiergens T.S., Engel J., Jung A., Hermeking H., Grunewald T.G.P., Kirchner T. (2018). PBX3 Is Part of an EMT Regulatory Network and Indicates Poor Outcome in Colorectal Cancer. Clin. Cancer Res. J. Am. Assoc. Cancer Res..

[53] Abbaszadegan M.R., Bagheri V., Razavi M.S., Momtazi A.A., Sahebkar A., Gholamin M. (2017). Isolation, identification, and characterization of cancer stem cells: A review. J. Cell Physiol..

[54] Xu X., Cai N., Bao Z., You Y., Ji J., Liu N. (2017). Silencing Pre-B-cell leukemia homeobox 3 decreases the proliferation of human glioma cells in vitro and in vivo. J. Neurooncol..

[55] Pan C., Gao H., Zheng N., Gao Q., Si Y., Zhao Y. (2017). MiR-320 inhibits the growth of glioma cells through downregulating PBX3. Biol. Res..

[56] Wang G., Wang J., Zhao H., Wang J., To S.S.T. (2015). The role of Myc and let-7a in glioblastoma, glucose metabolism and response to therapy. Arch. Biochem. Biophys..

[57] Li H., Sun G., Liu C., Wang J., Jing R., Wang J., Zhao X., Xu X., Yang Y. (2017). PBX3 is associated with proliferation and poor prognosis in patients with cervical cancer. OncoTargets Ther..

[58] Jo V.Y., Antonescu C.R., Dickson B.C., Swanson D., Zhang L., Fletcher C.D.M., Demicco E.G. (2019). Cutaneous Syncytial Myoepithelioma is Characterized by Recurrent EWSR1-PBX3 Fusions. Am. J. Surg. Pathol..

[59] Panagopoulos I., Gorunova L., Bjerkehagen B., Heim S. (2015). Fusion of the genes EWSR1 and PBX3 in retroperitoneal leiomyoma with t(9;22)(q33;q12). PLoS ONE.

[60] Yun S., Kim S.H., Cho H.S., Choe G., Lee K.S. (2019). EWSR1-PBX3 fused myoepithelioma arising in metatarsal bone: Case report and review of the literature. Pathol. Int..

[61] Hill R., Madureira P.A., Ferreira B., Baptista I., Machado S., Colaco L., Dos Santos M., Liu N., Dopazo A., Ugurel S. (2017). TRIB2 confers resistance to anti-cancer therapy by activating the serine/threonine protein kinase AKT. Nat. Commun..

[62] Morgan R., El-Tanani M., Hunter K.D., Harrington K.J., Pandha H.S. (2017). Targeting HOX/PBX dimers in cancer. Oncotarget.

[63] Yan B., Wang H., Tan Y., Fu W. (2019). microRNAs in Cardiovascular Disease: Small Molecules but Big Roles. Curr. Top Med. Chem..

[64] van Zandwijk N., Pavlakis N., Kao S.C., Linton A., Boyer M.J., Clarke S., Huynh Y., Chrzanowska A., Fulham M.J., Bailey D.L. (2017). Safety and activity of microRNA-loaded minicells in patients with recurrent malignant pleural mesothelioma: A first-in-man, phase 1, open-label, dose-escalation study. Lancet Oncol..

[65] Watt G.F., Scott-Stevens P., Gaohua L. (2019). Targeted protein degradation in vivo with Proteolysis Targeting Chimeras: Current status and future considerations. Drug Discov. Today Technol..

